# Aortic valve calcification across stages of dysglycemia in middle-aged individuals from the general population

**DOI:** 10.1186/s12933-025-02634-7

**Published:** 2025-03-05

**Authors:** Anne Wang, Carl Johan Östgren, Anna Norhammar, David Kylhammar, Tomas Jernberg, Lars Lind, Stefan Söderberg, Anders Blomberg, Gunnar Engström, Göran Bergström, Magnus Settergren, Bahira Shahim

**Affiliations:** 1 Department of Medicine Solna, Karolinska Institutet, 171 76 Stockholm, Sweden; 2 Department of Cardiology, Heart, Vascular and Neuro Theme, Karolinska University Hospital, Stockholm, Sweden; 3 Department of Health, Medicine and Caring Sciences, Linköping University, Linköping, Sweden; 4 Centre of Medical Image Science and Visualization, Linköping University, Linköping, Sweden; 5 Capio St Göran Hospital, Stockholm, Sweden; 6 Department of Clinical Physiology, Linköping University, Linköping, Sweden; 7 Wallenberg Centre for Molecular Medicine, Linköping University, Linköping, Sweden; 8 Department of Clinical Sciences, Karolinska Institutet, Danderyd Hospital, Stockholm, Sweden; 9 Department of Medical Sciences, Uppsala University, Uppsala, Sweden; 10 Department of Public Health and Clinical Medicine, Umeå University, Umeå, Sweden; 11 Department of Clinical Science in Malmö, Lund University, Lund, Sweden; 12 Department of Molecular and Clinical Medicine, Institute of Medicine, Sahlgrenska Academy, University of Gothenburg, Gothenburg, Sweden; 13 Department of Clinical Physiology, Sahlgrenska University Hospital, Gothenburg, Region Västra Götaland Sweden

**Keywords:** Dysglycemia, Prediabetes, Diabetes, Aortic valve calcification, Aortic stenosis, Epidemiology, Prevention, HbA1c, Fasting glucose

## Abstract

**Background:**

Aortic valve calcification (AVC) is an underlying pathophysiological mechanism in aortic stenosis, which shares many risk factors with diabetes. However, the association between dysglycemia and early stages of AVC remains unclear. The aim was to examine the associations between stages of dysglycemia and signs of AVC among middle-aged individuals from the general population.

**Methods:**

This was a cross-sectional study from the Swedish CArdioPulmonary bioImage Study (SCAPIS) randomly enrolling 30,154 middle-aged men and women from six study sites in Sweden between 2013 and 2018. Glycemic status was based on the World Health Organization criteria (fasting blood glucose and/or HbA1c) and questionnaire-based answers on previous diseases and categorized as normoglycemia, prediabetes, newly detected diabetes and known diabetes. AVC was assessed on cardiac computed tomography (CT) and defined as evident or not.

**Results:**

Of 29,331 individuals with data on glycemic status and AVC available, mean age was 57.5 years and normoglycemia was present in 76%, prediabetes in 16%, newly detected diabetes in 3% and known diabetes in 5%. The prevalence of AVC increased progressively across glycemic categories, particularly in males (8%, 11%, 14% and 17%; *P* < 0.01) compared to females (5%, 6%, 8% and 9%; *P* < 0.01). There was an association with AVC already in the early stages of dysglycemia; prediabetes (OR 1.16, 95% CI 1.02–1.31), newly detected diabetes (1.34 [1.05–1.71]) and known diabetes (1.61 [1.34–1.93]) after adjusting for age, sex, smoking, study site, low density lipoprotein-cholesterol and hypertension.

**Conclusions:**

In this large, contemporary, and randomly selected population of middle-aged individuals, prediabetes, newly detected diabetes and known diabetes were all associated with CT-detected AVC. Further studies are warranted to investigate if managing dysglycemia, even in its early stages, may help slow down AVC progression.

**Graphical abstract:**

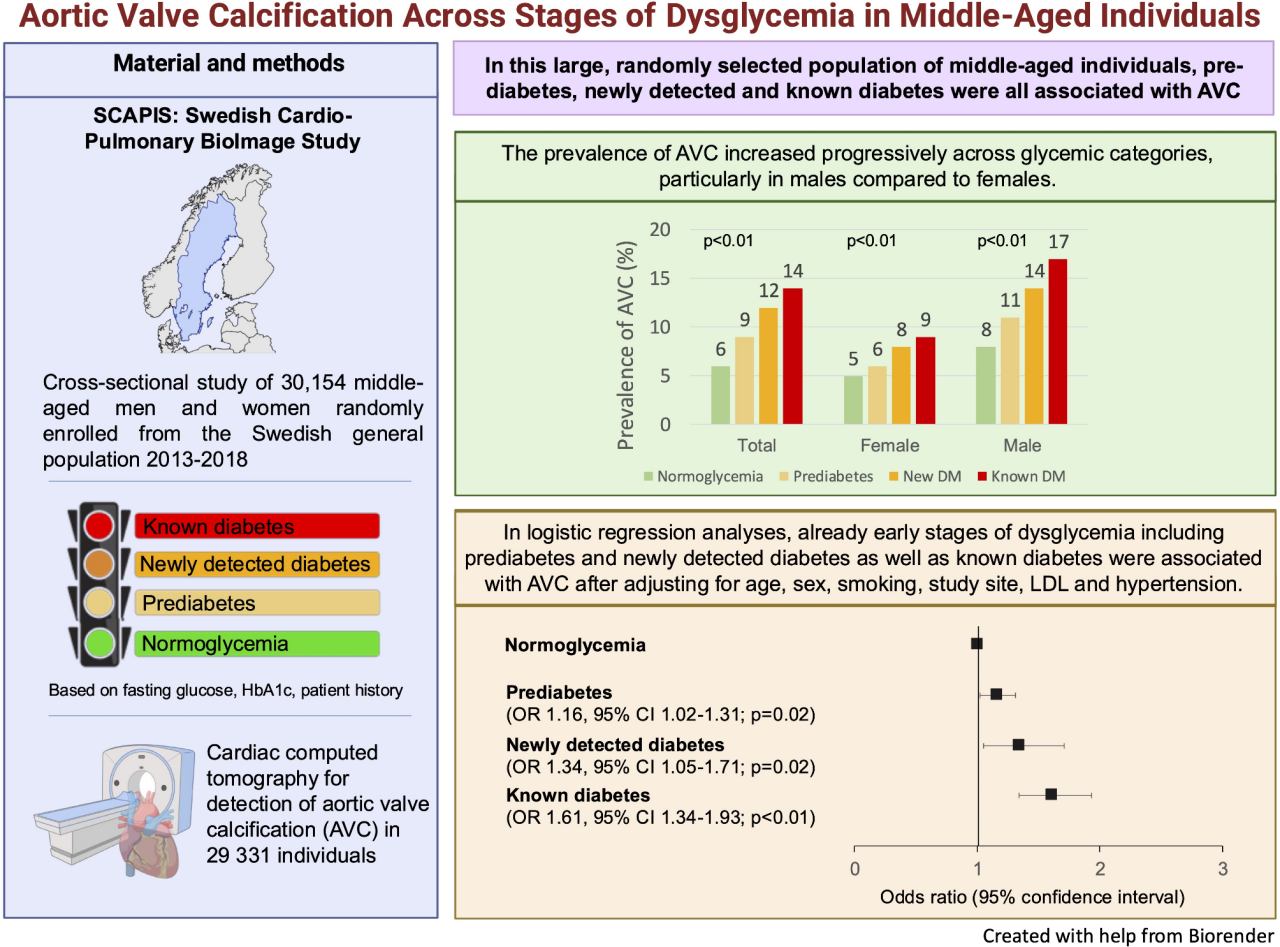

**Supplementary Information:**

The online version contains supplementary material available at 10.1186/s12933-025-02634-7.

## Introduction

Degenerative aortic stenosis (AS) is the most common valvular heart disease requiring intervention in developed countries, and its prevalence is expected to rise partly as a consequence of the aging population [1–3]. If left untreated, symptomatic severe AS is associated with a 2-year mortality rate as high as 50% [4]. Currently, there are no medical therapies available to reduce the disease burden or treat the underlying pathologies, making aortic valve replacement the only treatment option [2].

In the early stages of aortic valve calcification (AVC), inflammatory cells initiate a fibrotic process that stiffens the valve, leading to calcific masses within the aortic cusps. As the calcific masses grow, they can cause hemodynamic obstruction, leading to AS [5, 6]. The early stages of AVC are common in the elderly and have been linked to increased cardiovascular morbidity and mortality [7, 8]. Risk factors for AVC/AS appear to overlap partially with those for diabetes including smoking, obesity, hypertension and hyperlipidemia. Therefore, dysglycemia has been proposed as a possible mediator that accelerates the fibrocalcific process [9]. However, most studies have investigated dysglycemia and AS rather than AVC regardless of hemodynamic obstructions. AVC can be detected already in its early stages using cardiac computed tomography (CT), which may offer an optimal window to initiate preventive measures to slow disease progression. Previous studies on diabetes and AVC have been limited by small sample sizes, elderly populations, the use of echocardiography instead of CT for detecting or quantifying calcification and have shown inconclusive results [7, 10–13]. Additionally, the full spectrum of dysglycemia, including prediabetes, has rarely been studied in relation to AVC.

To better characterize the relationship between different levels of dysglycemia and AVC, we performed a comprehensive analysis using the Swedish CArdioPulmonary BioImage Study (SCAPIS), which prospectively included randomly selected middle-aged individuals from the general population, who underwent extensive cardiac CT scans and dysglycemia testing.

## Methods

### Study population

SCAPIS is a population-based cohort comprising 30,154 men and women aged 50–64 years who were randomly recruited from six university sites in Sweden (Gothenburg, Linköping, Lund/Malmö, Stockholm, Umeå, Uppsala). The main objective of SCAPIS is to characterize middle-aged men and women using advanced imaging methods and omics technologies to identify and treat cardiopulmonary disease [14]. Study participants were excluded if they could not understand written and spoken Swedish for the informed consent. Clinical examination included anthropometric measures, electrocardiography and blood pressure. Questionnaires were used to obtain information on medical history (such as known diabetes, hypertension and hyperlipidemia), pharmacological treatment, socioeconomic factors and tobacco use. Smoking status was defined as current or former smoker and never-smoker, respectively. Blood samples were collected after an overnight fast and sent for analysis immediately according to local hospital routine. Creatinine was used to calculate the estimated glomerular filtration rate (eGFR) [15].

### Glycemic status

Study participants were categorized based on fasting blood glucose and/or HbA1c at baseline according to the World Health Organization’s (WHO) classification: normoglycemic (glucose < 6.1 mmol/L and HbA1c < 42 mmol/mol), prediabetes (impaired fasting glucose (IFG) 6.1–6.9 mmol/L and/or elevated HbA1c 42–47 mmol/mol and NO on self-reported diabetes on the questionnaire), newly detected diabetes (glucose ≥7 mmol/L and/or HbA1c ≥48 mmol/mol and NO on self-reported diabetes on the questionnaire) or self-reported diabetes on the questionnaire. In addition, prediabetes was further categorized based on diagnosis either by IFG (fasting glucose 6.1–6.9 mmol/L) or elevated HbA1c (42–47 mmol/mol) and were studied separately.

As a secondary exposure, dysglycemia defined according to the American Diabetes Association’s (ADA) classification was used with a difference in definitions of normoglycemia (glucose < 5.6 mmol/L and HbA1c < 39 mmol/mol) and prediabetes (IFG 5.6–6.9 mmol/L or elevated HbA1c 39–47 mmol/mol) [16]. This additional classification was also made based on fasting blood glucose levels, HbA1c and results from questionnaires. The reason for using both WHO and ADA’s definition was to strengthen the generalizability of the results, given that the different definitions are used in different parts of the world.

### Cardiac imaging

Of 30,154 study participants, 29,613 underwent cardiac CT, which was performed with a dual-source CT scanner with a Stellar Detector (Somatom Definition Flash, Siemens Medical Solution, Forchheim, Germany). A standardized sequential image acquisition workflow was developed to allow for imaging of heart and lungs in one session. CT images for AVC were obtained using electrocardiogram-gated non-contrast imaging at 120 kV. At each respective site, the CT scanners were maintained using identical software and hardware, and images were read by experienced radiologists within the SCAPIS study. The presence of any AVC was reported as yes/no by the study readers (comprised of 36 trained thoracic radiologists or cardiologists) with between 1 and > 10 years of training in reading coronary computed tomography angiography. This was entered into the SCAPIS central case report forms consecutively. Additionally, coronary artery calcification (CAC) was assessed in which the calcium content was measured in each coronary artery and subsequently summed to provide a CAC score in Agatston units. For the current study, a CAC score > 0 was defined as presence of CAC.

### Statistical analysis

Data were analyzed without imputations given the low number of missing values. Continuous variables are presented as mean (standard deviation) and compared by ANOVA and categorical variables are presented as numbers (percentages) and compared by Pearson’s chi-squared test. Logistic regression models were used to estimate odds ratio (OR) to evaluate the association between glycemic status and AVC. Each variable was run in an unadjusted model as well as in a multivariable model adjusted for age, sex, smoking, study site, low density lipoprotein (LDL)-cholesterol and hypertension. Covariates were chosen after literature review (Supplementary file 1: Table 1) and careful consideration following the construction of a Directed Acyclical Graph to outline the inter-relations between the variables (Supplementary file 1: Fig. 1). As part of a sensitivity analysis and given the close link between body mass index (BMI), insulin resistance and dysglycemia, BMI was added in addition to the previous covariates in the multivariable model. Moreover, logistic regression analysis with an interaction term (glycemic status times sex) was performed in unadjusted and adjusted models to assess whether there was any interaction between dysglycemia and sex regarding AVC. A two-sided *P*-value < 0.05 was considered significant. Analyses were performed in R version 4.3.0 and STATA/MP 18.0.

## Results

### Baseline characteristics

Out of 30,154 study participants enrolled, 30,120 had data on glycemic status available whereof 29,331 also had data available on AVC. Baseline characteristics by glycemic status are outlined in Table 1. Briefly, mean age was 57.5 years and 51% were females. Normoglycemia was present in 22,507 (76%), prediabetes in 4,642 (16%), newly detected diabetes in 810 (3%) and known diabetes in 1372 (5%) (Supplementary file 1: Fig. 2A). Among prediabetes, 3,436 (72%) were detected by IFG only, 725 (15%) by elevated HbA1c only and 443 (9%) by both IFG and elevated HbA1c. As expected, increasing severity of dysglycemia was associated with increasing age, higher proportion of males, more smokers, higher BMI and more previous cardiovascular disease. Using ADAs definition, prediabetes was found in a substantially higher proportion (11,642 (39%); normoglycemia in 15,498 (53%); Supplementary file 1: Fig. 2B).

**Table 1:** Baseline characteristics by glycemic status (based on fasting glucose and HbA1c levels according to the World Health Organization definition)

Characteristic	Overall, *N* = 29,331	Normoglycemia, *N* = 22,507	Prediabetes, *N* = 4642	New DM, *N* = 810	Known DM, *N* = 1,372	*P*-value
Age (years)	57.5 (4.3)	57.2 (4.3)	58.2 (4.3)	58.8 (4.1)	59.0 (4.1)	< 0.001
Female	14,971 (51%)	12,017 (54%)	2134 (46%)	322 (40%)	498 (36%)	< 0.001
Birthplace Sweden	24,059 (84%)	18,662 (85%)	3773 (83%)	616 (79%)	1008 (76%)	< 0.001
University degree	12,915 (45%)	10,417 (47%)	1788 (40%)	298 (39%)	412 (31%)	< 0.001
Smoking	3720 (13%)	2623 (12%)	732 (16%)	132 (17%)	233 (18%)	< 0.001
BMI (kg/m^2^)	27.0 (4.5)	26.4 (4.1)	28.4 (4.8)	30.2 (5.3)	30.1 (5.1)	< 0.001
Waist Hip ratio	0.92 (0.09)	0.91 (0.09)	0.94 (0.09)	0.97 (0.09)	0.99 (0.09)	< 0.001
Systolic blood pressure (mmHg)	126 (17)	125 (17)	129 (17)	133 (18)	131 (15)	< 0.001
Hypertension	6429 (23%)	4049 (19%)	1340 (30%)	267 (35%)	773 (59%)	< 0.001
Hyperlipidemia	3316 (12%)	1910 (9%)	627 (14%)	135 (18%)	644 (49%)	< 0.001
Myocardial Infarction	422 (1.5%)	197 (0.9%)	126 (2.8%)	18 (2.4%)	81 (6.2%)	< 0.001
Stroke	402 (1.4%)	255 (1.2%)	89 (2.0%)	17 (2.3%)	41 (3.2%)	< 0.001
Heart Failure	134 (0.5%)	69 (0.3%)	31 (0.7%)	7 (0.9%)	27 (2.1%)	< 0.001
Coronary artery calcification	12,339 (42%)	8682 (39%)	2240 (48%)	477 (59%)	940 (69%)	< 0.001
Chronic kidney dysfunction*	665 (2.3%)	488 (2.2%)	104 (2.2%)	15 (1.9%)	58 (4.2%)	< 0.001
eGFR (ml/min/1.73 m^2^)	85 (12)	84 (12)	86 (12)	87 (13)	88 (14)	< 0.001
LDL-cholesterol (mmol/L)	3.44 (0.97)	3.52 (0.94)	3.32 (0.95)	3.38 (1.03)	2.63 (0.93)	< 0.001
hs-CRP (mg/dl)	2.11 (4.17)	1.89 (3.57)	2.64 (4.53)	3.81 (10.52)	2.96 (4.91)	< 0.001
Fasting glucose (mmol/L)	5.61 (1.12)	5.24 (0.48)	6.21 (0.44)	7.72 (1.73)	8.33 (2.71)	< 0.001
HbA1c (mmol/mol)	36.5 (6.4)	34.9 (3.0)	38.0 (4.0)	45.2 (12.6)	53.2 (14.4)	< 0.001
Insulin treatment	348 (33%)	0 (NA%)	0 (NA%)	0 (NA%)	348 (33%)	
Diabetes duration (years)	10 (10)	NA (NA)	NA (NA)	NA (NA)	10 (10)	

BMI = Body mass index; eGFR = estimated glomerular filtration rate; LDL = low density lipoprotein; hs-CRP = high sensitivity C-reactive protein; HbA1c = glycated hemoglobin A1c

*Defined as eGFR < 60 ml/min/1.73 m^2^ according to the Kidney Disease: Improving Global Outcome (KDIGO) staging

### Prevalence of AVC in dysglycemia

**Fig. 1:**
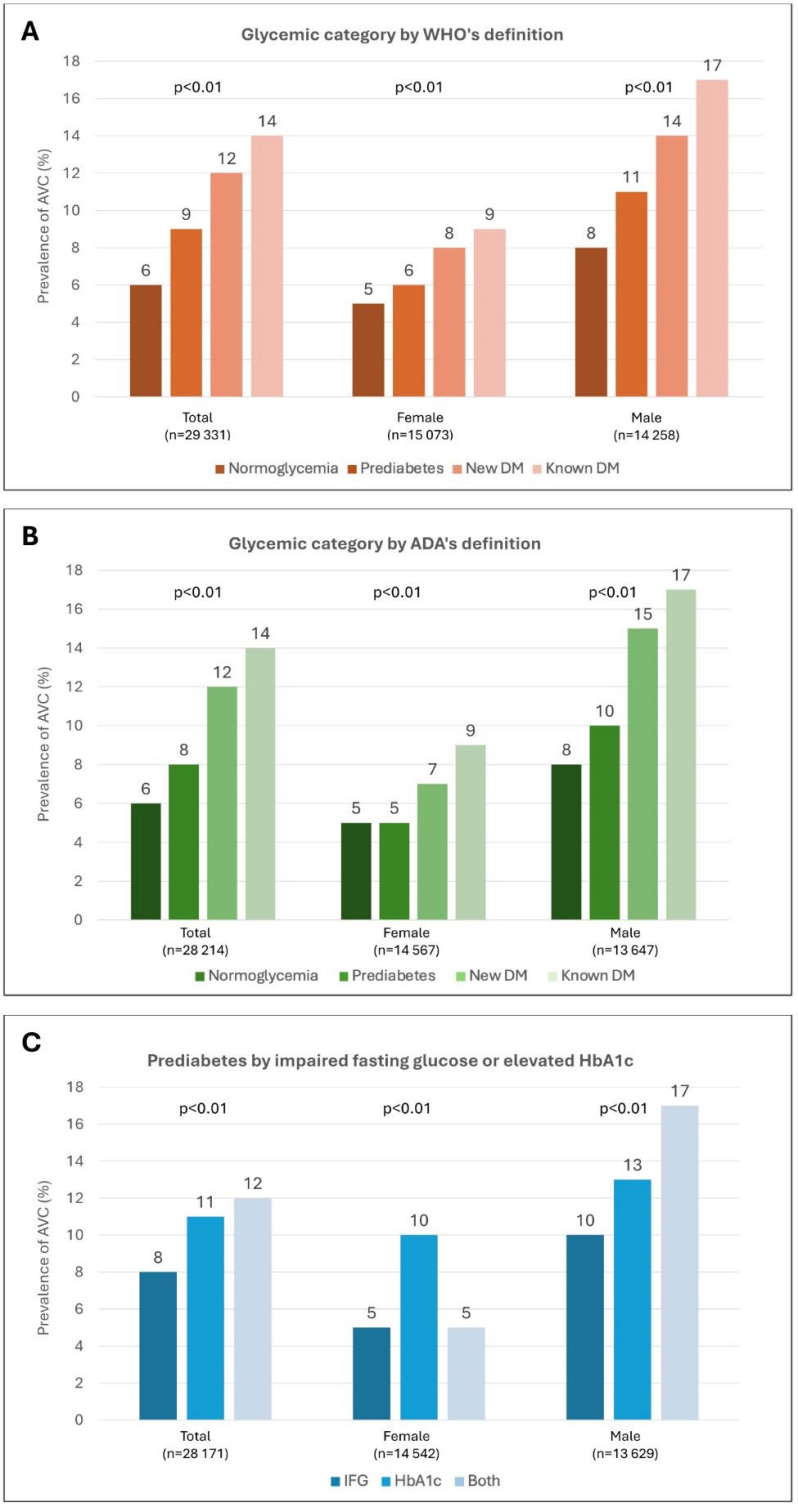
Prevalence of aortic valve calcification by glycemic category defined using fasting glucose and HbA1c; (**A**) by the World Health Organization’s definition; (**B**) by the American Diabetes Association’s definition; (**C**) By prediabetes (WHO's definition) categorized as impaired fasting glucose, elevated HbA1c and both combined. *WHO* World Health Organization; *ADA* American Diabetes Association;*DM* diabetes mellitus; *IFG* impaired fasting glucose; *HbA1c*   glycated hemoglobin A1c

In the total cohort, AVC was found in 2,123 (7%) study participants. The prevalence of AVC increased by glycemic category, from 6% in normoglycemia to 9% in prediabetes, 12% in newly detected diabetes and 14% in known diabetes (Fig. 1A). When stratified by sex, the prevalence of AVC by increasing glycemic category was substantially higher in males (8%, 11%, 14% and 17% in males (*P* < 0.01)) compared to females (5%, 6%, 8% and 9% (*P* < 0.01)). Similar results were shown using the ADA definition (Fig. 1B). The prevalence of AVC was lower in prediabetes detected with IFG (8%) compared to those with elevated HbA1c (11%) or both IFG and elevated HbA1c (12%; *P* < 0.001), with somewhat different pattern between men and women (Fig. 1C).

### Association between dysglycemia and AVC

**Fig. 2:**
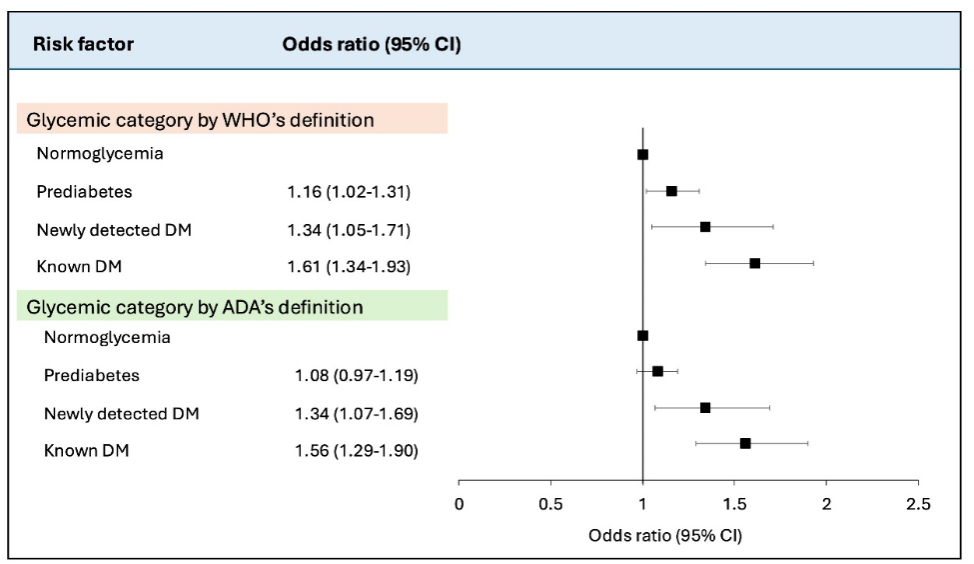
Association between different levels of dysglycemia and aortic valve calcification adjusted for age, sex, smoking, study site, low-density lipoprotein cholesterol and hypertension. *WHO* World Health Organization; *ADA* American Diabetes Association;*DM* diabetes mellitus

**Fig. 3:**
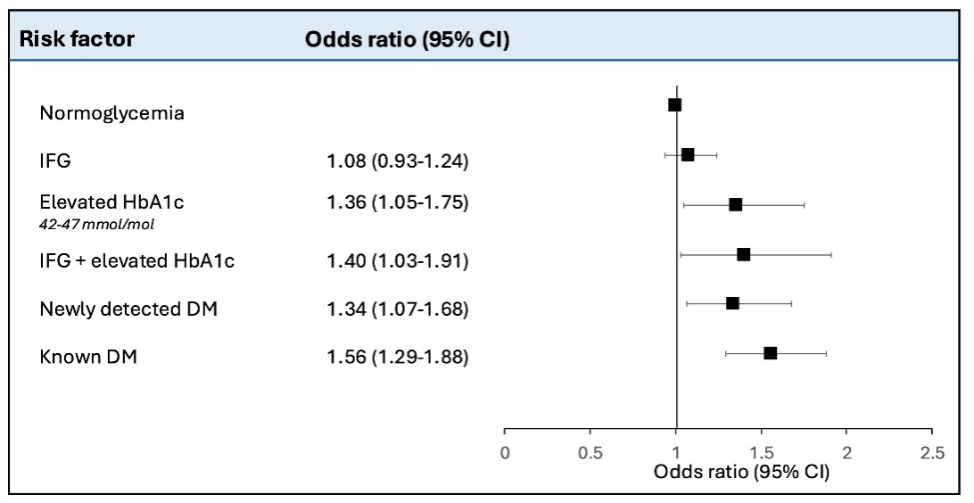
Association between different levels of dysglycemia including prediabetes categorized as (**a**) impaired fasting glucose; (**b**) elevated HbA1c 42–47 mmol/mol; (**c**) impaired fasting glucose + elevated HbA1c by the World Health Organization’s definition. Adjustments made for age, sex, smoking, study site, low-density lipoprotein cholesterol and hypertension. *IFG* impaired fasting glucose; *HbA1c*   glycated hemoglobin A1c; *DM* diabetes mellitus

In unadjusted analyses for dysglycemia according to the WHO definition, prediabetes (OR 1.47, 95% CI 1.31–1.64), newly detected diabetes (1.98 [1.58–2.45]) and known diabetes (2.47 [2.09–2.89]) were all associated with AVC compared with normoglycemia (Supplementary file 1: Fig. 3). Similar results were shown for the ADA definition. Following adjustment for age, sex, smoking, study site, LDL-cholesterol and hypertension, the association with AVC for all dysglycemic categories remained significant: prediabetes (OR 1.16, 95% CI 1.02–1.31; *P* = 0.02), newly detected diabetes (1.34 [1.05–1.71]) and known diabetes (1.61 [1.34–1.93]) compared with individuals with normoglycemia. These associations remained similar although slightly attenuated for prediabetes when dysglycemia was categorized according to the ADA definition (Fig. 2). In analyses by different prediabetes classes, all categories were associated with AVC in the unadjusted model (IFG: OR 1.29, 95% CI 1.12–1.48 and Elevated HbA1c: OR 1,87, 95% CI 1.46–2.36; Supplementary file 1: Fig. 4). In fully adjusted analyses, IFG was no longer associated with AVC (OR 1.08, 95% CI 0.93–1.24; *P* < 0.01) but elevated HbA1c (1.36 [1.05–1.75]) and both elevated HbA1c and IFG (1.40 [1.03–1.91]) remained significant (Fig. 3) compared with normoglycemia. An interaction analysis for sex was performed which did not show any significant association (Supplementary file 1: Table 2).

In a sensitivity analysis adding BMI as a covariate to the multivariable model, diabetes remained significant (OR 1.48, 95% CI 1.23–1.79) but the association with AVC for the early stages of dysglycemia was attenuated (prediabetes: 1.10 [0.97–1.25] and newly detected diabetes 1.23 [0.96–1.57]) (Supplementary file 1: Table 3). The similar trend was seen for the ADA definition.

## Discussion

To the best of our knowledge, the present analysis represents the largest and most well-characterized study to date for assessing the relationship between various levels of dysglycemia and CT-detected AVC among randomly selected, middle-aged individuals from the general population. There were three main findings: (1) the prevalence of AVC increased progressively across the dysglycemic categories of prediabetes, newly detected diabetes and known diabetes; (2) there was an association already in the early stages of dysglycemia with AVC; (3) and among individuals with prediabetes, AVC was more commonly detected when using HbA1c compared to IFG.

### Prevalence of AVC

Previous studies have shown a higher prevalence of AS in those with compared to without diabetes and vice versa [17, 18], but little is known about the prevalence of AVC across the different stages of dysglycemia, especially in middle-aged populations. In this analysis, the prevalence of AVC was more than twice as high in individuals with already known diabetes, particularly among men, in whom the prevalence of AVC was 17% compared to 8% in those without diabetes. AVC was also more common in those with newly detected diabetes (present in 14%). Notably, the prevalence of AVC was higher already in those with prediabetes, especially in individuals with elevated HbA1c. These findings suggest that even early stages of hyperglycemia may contribute to the pathophysiology of AVC and that HbA1c, which reflects chronic glucose exposure, may be a better marker for identifying those at risk of AVC than fasting glucose. IFG may simply not lead to a hyperglycemic state of sufficient duration to cause sclerosis. Given that elevated HbA1c compared to IFG represent overlapping but distinct abnormalities in the glucose metabolism, studying these entities separately provides clinically meaningful insights into the pathophysiology of AVC and suggest HbA1c rather than fasting glucose as a potential screening tool for future initiatives to detect AVC. Interestingly, in a prospective study comprising patients undergoing surgery for valvular heart disease and matched controls, diabetes but neither IFG nor impaired glucose tolerance were associated with AS requiring valve replacement surgery [18]. This could suggest that while different levels of dysglycemia may be important for lesion formation and early calcification, only diabetes and not prediabetes remains as a risk in advanced aortic stenosis where other factors may have a greater influence on hemodynamic disturbances.

AVC has previously been associated heavily with increasing age and the presence of any AVC in men before 60 and women before 65 years of age was very uncommon in a recent study from the Multi-Ethnic Study of Atherosclerosis (MESA) [19]. As such, the presence of premature AVC in a middle-aged population such as ours should be considered abnormal. Apart from AVC being associated with increasing cardiovascular morbidity and mortality [7], it is also strongly associated with the long-term risk of developing AS [19]. To date, there are no medical therapies available for reducing the calcification process in the aortic valves and limiting progression from AVC to AS. However, recent evidence has shown promising results for sodium-glucose cotransporter-2 (SGLT2) protein and inhibition in AS. First of all, the SGLT2 gene and protein were hyper-expressed in human cardiomyocytes in patients with severe AS of low-flow low-gradient type [20]. Second, in a cohort study following 311 patients with diabetes for a median of two years after transcatheter aortic valve implantation, the use of SGLT2 inhibitors was associated with a more favorable cardiac remodeling as well as improved cardiovascular outcomes [21]. This indicates that SGLT2i has the potential to improve the course of disease in the later stages of AS in patients with diabetes and introduces a long-awaited therapeutic avenue in AS in need of further study. Identifying individuals with AVC and dysglycemia could guide the selection of participants for future clinical trials investigating whether optimal glucose management may also slow down the disease in the early stages including progression of AVC.

### Dysglycemia as a risk factor for AVC

Dysglycemia was associated with AVC even in its early stages. Prior studies investigating this relationship have primarily focused on established diabetes, involved small sample sizes and reported inconsistent findings. A study from the Cardiovascular Health Study, which included elderly individuals (> 65 years), found that smoking, hypertension and lipid levels, but not diabetes, were associated with echocardiography-detected AVC [10]. In contrast, the Epidemiology of Coronary Artery Calcification study demonstrated a significant association between diabetes and AVC, as determined by CT in 262 individuals [22]. Similarly, the MESA cohort study showed that diabetes was associated with CT-detected AVC in 6,780 men and women [11]. The present study expands on previous knowledge by investigating dysglycemia as a continuous spectrum rather than a binary classification of diabetes or not. The attenuated results for early stages of dysglycemia and AVC following adjustment of BMI indicates that BMI constitutes a substantial part of the pathophysiology driving insulin resistance and subsequently leading to dysglycemia. Dysglycemia and obesity often coexists and previous studies show that up to 85% of people with type 2 diabetes are overweight or obese, highlighting the complex interplay between dysglycemia and obesity in the modulation of cardiovascular disease [23, 24]. The results are in line with data from the Jackson Heart Study (*n* = 1,664 individuals) in which diabetes, but not prediabetes, was associated with abdominal aortic calcification following adjustment for several confounders including BMI [25]. Moreover, the results were slightly attenuated when using ADA’s broader definition for prediabetes, indicating that while dysglycemia should be viewed as a spectrum, there is likely a threshold closer to the WHO definition for when it accelerates the risk for AVC.

Individuals with diabetes were more often men and had in general more cardiovascular risk factors such as hypertension, smoking and hyperlipidemia, all of which are associated with AVC [13, 26]. Additionally, increasing dysglycemia has been associated with a higher prevalence of chronic kidney disease and CAC, conditions that are also more common in individuals with AVC/AS [22, 27]. These findings suggest that a systemic fibrotic process in AVC may contribute to progressive valve stiffness [5]. While male sex is considered a risk for AVC [28], sex did not modulate the effect of dysglycemia on AVC in the present study. The elevated risk of AVC in individuals with dysglycemia may be related to a worse overall risk factor profile, which could accelerate the calcification process. In a study from the Swedish National Diabetes registry, patients with type 2 diabetes with an increasing number of cardiovascular risk factors (higher systolic blood pressure, elevated BMI, elevated eGFR) were at a higher risk of AS [29]. However, even patients with diabetes and optimal control of cardiovascular risk factors (i.e. no smoking, HbA1c, systolic and diastolic blood pressure, LDL-cholesterol and albuminuria within target range) remained at a higher risk compared to their counterparts without diabetes, indicating that traditional risk factors alone may not fully explain the association between diabetes and AVC/AS [29]. It is also possible that dysglycemia may directly affect the aortic valves. Type 2 diabetes have been shown to augment the expression of pro-inflammatory proteins in patients with severe AS [30]. Additionally, dysglycemia enhances oxidative stress and glycosylation of proteins and lipids as well as activation of coagulation factors, further contributing to inflammation and calcification [9]. Moreover, chronic hyperglycemia has been associated with an accelerated formation of advanced glycation end products (AGE), which has been attributed to vascular calcification in patients with diabetes [31, 32]. In a prospective study comprising patients with severe AS with (*n* = 50) and without diabetes (*n* = 76) undergoing aortic valve replacement, more AGEs were found accumulated in the stenotic aortic valves in patients with diabetes. In addition, the extent of AGE accumulation correlated with AS severity, suggesting that AGE may be considered as a key pathophysiological mechanism driving AS progression in patients with diabetes [33]. Finally, although dysglycemia is associated with AVC, studies have indicated that traditional cardiovascular risk factors only contribute up to a third of the risk for AS [34]. This indicates that other factors, yet unknown, may play a more important role in the pathophysiology for valvular calcification.

## Limitations

Our study has some limitations. First of all, SCAPIS comprises mainly individuals born in Sweden, thus limiting the generalizability to other ethnicities. Second, AVC was only categorized as evident or not without further quantification of the extent of AVC with calcium scoring. Third, the definition of prediabetes was made based on HbA1c and fasting glucose. Oral glucose tolerance tests were not performed during the study, thus possibly missing individuals with impaired glucose tolerance as well as undetected diabetes. However, screening for diabetes in individuals with cardiovascular disease using HbA1c and/or fasting glucose is in line with the latest recommendations from the 2023 European Society of Cardiology’s guidelines on diabetes and cardiovascular disease [35]. Finally, the cross-sectional design lacks temporal information, as exposure and outcome are assessed at the same time, limiting the possibility to establish causality. Longitudinal studies are needed to further clarify the relationship between dysglycemia and AVC.

## Conclusions

Dysglycemia categorized as prediabetes, newly detected diabetes and known diabetes were all associated with CT-detected AVC in a large, contemporary and randomly selected population of middle-aged individuals. In a sensitivity analysis including BMI as a covariate, the association for prediabetes and newly detected diabetes were attenuated, indicating that BMI constitutes a major part of the underlying mechanism in early dysglycemia and may act as a defining factor in this association with AVC. Individuals with dysglycemia could be an appropriate target for future strategies to identify AVC and underlines the need for clinical trials investigating the potential of optimal glucose-lowering management in the prevention for AVC progression.

## Electronic supplementary material

Below is the link to the electronic supplementary material.

Supplementary Material 1.

## Data Availability

The data underlying this manuscript cannot be shared publicly for legal regulations related to the privacy of individuals that participated in the study. The data are available from the corresponding author (AW) and principal investigator (BS) on reasonable request.

## Abbreviations

ADA: American Diabetes Association
AS: Aortic stenosis
AVC: Aortic valve calcification
CT: Computed tomography
IFG: Impaired fasting glucose
SCAPIS: Swedish CardioPulmonary BioImage Study
WHO: World Health Organization
