# Supplementary material for: Aortic valve calcification across stages of dysglycemia in middle-aged individuals from the general population
Source: Cardiovasc Diabetol. 2025 Mar 5;24:105. doi: 10.1186/s12933-025-02634-7 (PMC11884113; doi:10.1186/s12933-025-02634-7)
Supplement: Supplementary file 1 — Supplementary Material 1. [file 12933_2025_2634_MOESM1_ESM.docx]

***Supplementary data:***

**Aortic Valve Calcification Across Stages of Dysglycemia in Middle-Aged Inidviudals from the General Population**

*Short title: Dysglycemia and aortic valve calcification*

Anne Wang MD, PhD^a, b^, Carl Johan Östgren, MD, PhD^c, d^, Anna Norhammar, MD, PhD^a, e^, David Kylhammar, MD, PhD^c, d, f^, Tomas Jernberg^g^ MD, PhD, Lars Lind, MD, PhD^k^, Stefan Söderberg, MD, PhD^l^, Anders Blomberg, MD, PhD^l^, Gunnar Engström, MD, PhD^m^, Göran Bergström, MD, PhD^n, o^, Magnus Settergren, MD, PhD^b^, Bahira Shahim MD, PhD^a, b^

^a^Department of Medicine Solna, Karolinska Institutet, Stockholm, Sweden

^b^Cardiology Unit, Heart, Vascular and Neuro Theme, Karolinska University Hospital, Stockholm, Sweden

^c^Department of Health, Medicine and Caring Sciences, Linköping University, Linköping, Sweden

^d^Department of Clinical Physiology, Linköping University, Linköping, Sweden

^e^Capio St Göran,

^f^Wallenberg Centre for Molecular Medicine, Linköping University, Linköping, Sweden

^g^Department of Clinical Sciences, Danderyd Hospital, Karolinska Institutet, Stockholm

^h^Department of Cardiology, Linköping University, Linköping, Sweden

^i^Centre of Medical Image Science and Visualization, Linköping University, Linköping, Sweden

^j^Cardiothoracic Unit, Heart, Vascular and Neuro Theme, Karolinska University Hospital, Stockholm, Sweden

^k^Department of Medical Sciences, Uppsala University, Uppsala, Sweden

^l^Department of Public Health and Clinical Medicine, Umeå University, Umeå, Sweden

^m^Department of Clinical Science in Malmö, Lund University, Sweden

^n^Department of Molecular and Clinical Medicine, Institute of Medicine, Sahlgrenska Academy, University of Gothenburg, Gothenburg, Sweden.

^o^Department of Clinical Physiology, Sahlgrenska University Hospital, Region Västra Götaland, Gothenburg, Sweden.

**Address for correspondence**

Anne Wang MD, PhD

Department of Medicine Solna

Karolinska Institutet

171 76 Stockholm

Sweden

E-mail: anne.wang.gottlieb@ki.se

Telephone: 08 123 700 00

Tables: 2 Figures: 4

**Supplementary data**

**Supplementary table 1. Studies on risk factors for aortic valve calcification**

|  | **First author, year** | **Study design and population** | **Conclusion** |
| --- | --- | --- | --- |
| Helsinki Aging Study (1) | Lindroos M, 1994 | Cross-sectional design  n=577 ≥55 years  AVC assessed on echocardiography | **Hypertension**, **age** and a low body mass index were independent predictors of AVC |
| Cardiovascular Health Study (2) | Stewart BF, 1997 | Cross-sectional design  n=5,201 ≥65 years  AVC assessed on echocardiography | **Age, male sex, smoking, history of hypertension, lipoprotein(a), LDL-cholesterol** were associated with AVC |
| Metabolic Syndrome and Diabetes Mellitus as Predictors of Aortic Valve Calcification in the Multi-Ethnic Study of Atherosclerosis (3) | Katz R, 2006 | Cross-sectional design  n=6,780, 45-84 years  AVC assessed on computed tomography | **The metabolic syndrome and diabetes** were associated with AVC. In terms of separate components of the metabolic syndrome, **elevated blood pressure, high triglycerides in men, and abdominal obesity** (ATP III criteria) in women and abdominal obesity (IDF criteria) in men were independently associated with AVC. |
| Aortic valve calcification: determinants and progression in the population (4) | Messika-Zeitoun D, 2007 | Longitudinal design  n=262, ≥60 years  AVC assessed on computed tomography | AVC was frequent in elderly and in men. AVC was associated with **diabetes, hypertension, higher BMI and serum glucose**. De novo AVC was associated with **higher LDL-cholesterol.** |
| Incidence and progression of aortic valve calcium in the Multi-ethnic Study of Atherosclerosis (MESA) (5) | Owens D, 2010 | Longitudinal design  n=5,880 aged 45-84 years  AVC assessed on computed tomography | Incident AVC risk was associated with **age, male gender, body mass index, current smoking**, and the use of both **antihypertensive and lipid-lowering medications** |
| The association between aortic valve calcification, cardiovascular risk factors, and cardiac size and function in a general population (6) | Khurrami L, 2021 | Cross-sectional design  n=1,300, 65-75 years  AVC assessed on computed tomography | In a multiple regression analysis, **age, sex, prior cardiovascular disease, smoking, and hypertension** were associated with AVC score. |
| Cross-sectional study of aortic valve calcification and cardiovascular risk factors in older Danish men (7) | Khurrami L, 2021 | Cross-sectional design  n=14,073 men, 60-74 years  AVC assessed on computed tomography | **Age, hypertension, obesity, known cardiovascular disease, serum-phosphate levels** were associated with AVC. |
| **Studies on medication usage and aortic valve calcification** | | | |
| A randomized trial of intensive lipid lowering therapy in calcific aortic stenosis (8) | Cowell S, 2005 | Randomized, double-blind, placebo-controlled trial  n=77 with calcific aortic stenosis  Atorvastatin vs. placebo | Intensive lipid-lowering therapy does not halt the progression of calcific aortic stenosis or induce its regression. |
| (Intensive Lipid Lowering with Simvastatin and Ezetimibe in Aortic Stenosis (SEAS) trial (9) | Rossebo A, 2008 | Randomized double-blind placebo-controlled trial  n=1,873 with mild-moderate aortic stenosis  Simvastatin + ezetimibe vs. placebo for 52 months | Simvastatin and ezetimibe did not reduce the composite outcome of combined aortic-valve events and ischemic events in patients with aortic stenosis. |
| Effect of Lipid lowering with  rosuvastatin on progression of aortic stenosis: results of the aortic stenosis progression  observation: measuring effects of rosuvastatin (ASTRONOMER) trial (10) | Chan KL, 2010 | Randomized double-blind placebo-controlled trial  n=269 with mild-moderate aortic stenosis  Rosuvastatin vs. placebo for a median of 3.5 years | Cholesterol lowering with rosuvastatin 40 mg did not reduce the progression of AS in patients with mild to moderate AS; thus, statins should not be used for the sole purpose of reducing the progression of AS. |

**Supplementary table 2.** Interaction analysis between sex (male sex used as reference) and dysglycemia for the association with aortic valve calcification, unadjusted and adjusted for age, study site, smoking, LDL-cholesterol and hypertension.

|  | **Unadjusted** | | | | **Adjusted** | | | | |
| --- | --- | --- | --- | --- | --- | --- | --- | --- | --- |
|  | **OR** | | **95% CI** | **p-value** | **OR** | | **95% CI** | **p-value** | |
|  | **WHO’s definition for dysglycemia** | | | | | | | | |
| Prediabetes*Male | 1.06 | 0.83-1.35 | | 0.62 | 1.06 | 0.82-1.36 | | | 0.67 |
| Newly detected diabetes*Male | 1.03 | 0.64-1.70 | | 0.90 | 1.04 | 0.62-1.77 | | | 0.88 |
| Known diabetes*Male | 1.14 | 0.80-1.67 | | 0.49 | 1.09 | 0.74-1.61 | | | 0.65 |
|  | **ADA’s definition for dysglycemia** | | | | | | | | |
| Prediabetes*Male | 1.28 | 0.96-1.75 | | 0.16 | 1.21 | 0.98-1.48 | | | 0.07 |
| Newly detected diabetes*Male | 1.23 | 0.79-1.90 | | 0.37 | 1.03 | 0.64-1.67 | | | 0.91 |
| Known diabetes*Male | 1.26 | 0.85-1.87 | | 0.26 | 1.19 | 0.79-1.79 | | | 0.41 |

WHO=World Health Organization; ADA=American Diabetes Association

**Supplementary table 3.** Sensitivity analysis for the association between dysglycemia and aortic valve calcification by logistic regression. Adjusted for age, sex, study site, smoking, LDL-cholesterol, hypertension **and BMI**.

|  | OR (95% CI) | p-value |
| --- | --- | --- |
|  | WHO’s definition for dysglycemia | |
| Normoglycemia | 1.00 (ref) |  |
| Prediabetes | 1.10 (0.97-1.25) | 0.12 |
| Newly detected diabetes | 1.23 (0.96-1.57) | 0.11 |
| Known diabetes | 1.48 (1.23-1.79) | <0.01 |
|  | ADA’s definition for dysglycemia | |
| Normoglycemia | 1.00 (ref) |  |
| Prediabetes | 1.03 (0.93-1.15) | 0.52 |
| Newly detected diabetes | 1.22 (0.96-1.54) | 0.10 |
| Known diabetes | 1.42 (1.17-1.73) | <0.01 |

**Figure legends**

**Supplementary figure 1.** Directed Acyclical Graph for all sets of variables considered as potential confounders for the logistic regression analysis for dysglycemia and aortic valve calcification.

**Supplementary figure 2.** Distribution of glycemic status according to A) World Health Organization’s (WHO) and B) American Diabetes Association’s (ADA) definition for dysglycemia.

**Supplementary figure 3.** Unadjusted logistic regression for the association between different levels of dysglycemia and aortic valve calcification.
WHO=World Health Organization; ADA=American Diabetes Association; DM=Diabetes mellitus;

**Supplementary figure 4.** Unadjusted logistic regression for the association between different levels of dysglycemia including prediabetes categorized as a) impaired fasting glucose; b) elevated HbA1c; c) impaired fasting glucose + elevated HbA1c by World Health Organization’s definition.

IFG=Impaired fasting glucose; HbA1c=Glycated hemoglobin A1c; DM=Diabetes mellitus

Supplementary figure 1.

Supplementary figure 2.

Supplementary figure 3.

Supplementary figure 4.

References

1. Lindroos M, Kupari M, Valvanne J, Strandberg T, Heikkilä J, Tilvis R. Factors associated with calcific aortic valve degeneration in the elderly. Eur Heart J. 1994;15(7):865-70.

2. Stewart BF, Siscovick D, Lind BK, Gardin JM, Gottdiener JS, Smith VE, et al. Clinical factors associated with calcific aortic valve disease. Cardiovascular Health Study. J Am Coll Cardiol. 1997;29(3):630-4.

3. Katz R, Wong ND, Kronmal R, Takasu J, Shavelle DM, Probstfield JL, et al. Features of the metabolic syndrome and diabetes mellitus as predictors of aortic valve calcification in the Multi-Ethnic Study of Atherosclerosis. Circulation. 2006;113(17):2113-9.

4. Messika-Zeitoun D, Bielak LF, Peyser PA, Sheedy PF, Turner ST, Nkomo VT, et al. Aortic valve calcification: determinants and progression in the population. Arterioscler Thromb Vasc Biol. 2007;27(3):642-8.

5. Owens DS, Katz R, Takasu J, Kronmal R, Budoff MJ, O'Brien KD. Incidence and progression of aortic valve calcium in the Multi-ethnic Study of Atherosclerosis (MESA). Am J Cardiol. 2010;105(5):701-8.

6. Khurrami L, Møller JE, Dahl JS, Carter-Storch R, Christensen NL, Pareek M, et al. The association between aortic valve calcification, cardiovascular risk factors, and cardiac size and function in a general population. Int J Cardiovasc Imaging. 2021;37(2):711-22.

7. Khurrami L, Møller JE, Lindholt JS, Urbonaviciene G, Steffensen FH, Lambrechtsen J, et al. Cross-sectional study of aortic valve calcification and cardiovascular risk factors in older Danish men. Heart. 2021;107(19):1536-43.

8. Cowell SJ, Newby DE, Prescott RJ, Bloomfield P, Reid J, Northridge DB, et al. A randomized trial of intensive lipid-lowering therapy in calcific aortic stenosis. N Engl J Med. 2005;352(23):2389-97.

9. Rossebø AB, Pedersen TR, Boman K, Brudi P, Chambers JB, Egstrup K, et al. Intensive lipid lowering with simvastatin and ezetimibe in aortic stenosis. N Engl J Med. 2008;359(13):1343-56.

10. Chan KL, Teo K, Dumesnil JG, Ni A, Tam J, Investigators A. Effect of Lipid lowering with rosuvastatin on progression of aortic stenosis: results of the aortic stenosis progression observation: measuring effects of rosuvastatin (ASTRONOMER) trial. Circulation. 2010;121(2):306-14.
